# Wear Behavior Characterization of Hydrogels Constructs for Cartilage Tissue Replacement

**DOI:** 10.3390/ma14020428

**Published:** 2021-01-16

**Authors:** Saverio Affatato, Diego Trucco, Paola Taddei, Lorenzo Vannozzi, Leonardo Ricotti, Gilbert Daniel Nessim, Gina Lisignoli

**Affiliations:** 1 IRCSS Istituto Ortopedico Rizzoli, Laboratorio di Tecnologia Medica, 40136 Bologna, Italy; 2 IRCSS Istituto Ortopedico Rizzoli, SC Laboratorio di Immunoreumatologia e Rigenerazione Tissutale, 40136 Bologna, Italy; diego.trucco@santannapisa.it (D.T.); gina.lisignoli@ior.it (G.L.); 3 The BioRobotics Institute, Scuola Superiore Sant’Anna, Piazza Martiri della Libertà 33, 56127 Pisa, Italy; lorenzo.vannozzi@santannapisa.it (L.V.); leonardo.ricotti@santannapisa.it (L.R.); 4 Department of Excellence in Robotics & AI, Scuola Superiore Sant’Anna, Piazza Martiri della Libertà 33, 56127 Pisa, Italy; 5 Dipartimento di Scienze Biomediche e Neuromotorie, Università di Bologna, Via Belmeloro 8/2, 40126 Bologna, Italy; paola.taddei@unibo.it; 6 Department of Chemistry, Bar-Ilan Institute for Nanotechnology and Advanced Materials, Bar-Ilan University, Ramat Gan 52900, Israel; Gilbert.Nessim@biu.ac.il

**Keywords:** articular cartilage, knee arthroplasty, hydrogel, Raman spectroscopy, micro-CT, roughness measurements, knee simulator

## Abstract

This paper aims to characterize the wear behavior of hydrogel constructs designed for human articular cartilage replacement. To this purpose, poly (ethylene glycol) diacrylate (PEGDA) 10% *w*/*v* and gellan gum (GG) 1.5% *w*/*v* were used to reproduce the superior (SUP) cartilage layer and PEGDA 15% *w*/*v* and GG 1.5% *w*/*v* were used to reproduce the deep (DEEP) cartilage layer, with or without graphene oxide (GO). These materials (SUP and DEEP) were analyzed alone and in combination to mimic the zonal architecture of human articular cartilage. The developed constructs were tested using a four-station displacement control knee joint simulator under bovine calf serum. Roughness and micro-computer tomography (µ-CT) measurements evidenced that the hydrogels with 10% *w*/*v* of PEGDA showed a worse behavior both in terms of roughness increase and loss of uniformly distributed density than 15% *w*/*v* of PEGDA. The simultaneous presence of GO and 15% *w*/*v* PEGDA contributed to keeping the hydrogel construct’s characteristics. The Raman spectra of the control samples showed the presence of unreacted C=C bonds in all the hydrogels. The degree of crosslinking increased along the series SUP < DEEP + SUP < DEEP without GO. The Raman spectra of the tested hydrogels showed the loss of diacrylate groups in all the samples, due to the washout of unreacted PEGDA in bovine calf serum aqueous environment. The loss decreased along the series SUP > DEEP + SUP > DEEP, further confirming that the degree of photo-crosslinking of the starting materials plays a key role in determining their wear behavior. μ-CT and Raman spectroscopy proved to be suitable techniques to characterize the structure and composition of hydrogels.

## 1. Introduction

Knee osteoarthritis (OA), also known as degenerative joint disease, is typically the result of wear and tear and progressive loss of articular cartilage tissue [1]. Typical knee symptoms such as pain and loss of mobility increase as the OA progresses. When the symptoms can no longer be satisfactorily alleviated otherwise, knee prosthesis implant represents the ultimate step.

Modern total knee arthroplasty (TKA) consists of a femoral component, a tibial component, and a tibial platform/insert/meniscus. Femoral and tibial components are generally made of cobalt-chromium alloys (CoCr), whereas the tibial insert is made of ultra-high-molecular-weight polyethylene (UHMWPE). Total knee replacement (TKR) is one of the most consolidated prosthetic surgeries to restore the knee functions. Many types of prostheses have been used for TKA and TKR during the last 40 years, e.g., fixed or mobile knee prostheses, a total or unicondylar femoral component, cemented or cementless, etc. [2]. Anyway, this operation remains one of the most expensive prosthetic surgeries, constituting a significant burden for healthcare systems [3].

Articular cartilage is an avascular structure with poor self-healing properties and is characterized by distinct layers: superficial, middle, and deep zones and subchondral bone [4,5]. The superficial layer presents a dense concentration of collagen fibers aligned parallel to the surface, which reduces fluid permeability [6]. Both the middle and deep zones of the cartilage show a low content of vertically aligned collagen fibers, while chondrocytes show an elliptical shape along the fibers. The human knee articular cartilage superficial layer has the ability to support up to 74% of an average peak stress of 1.35 MPa, whereas the deep zone supports only 53% [7]. The main function of the articular cartilage in load-bearing diarthrodial joints is its ability to serve as a wear-resistant and low-friction surface [4]. Until now, articular cartilage regeneration has been challenging since different surgical procedures, like mosaic-plastic, micro-fractures, autologous chondrocyte implantation with or without matrix, as well as tissue engineering procedures, have not shown significant progress in cartilage regeneration [8,9].

Hydrogels represent an exciting class of biomaterials showing similarities with biological tissues in terms of hydration level and structure [10]. Hydrogels are water-swollen materials that can be considered optimal candidates for being developed into different three-dimensional (3D) shape molds [11] or into bio-printing processes [12]. Basically, hydrogels are three-dimensional cross-linked networks of hydrophilic polymer chains [13]. These polymeric networks can contain a quantity of water from several tens to thousands of times their dried mass. Several hydrogel-based systems currently constitute commercial products for various biomedical applications [14,15,16].

However, hydrogels alone cannot often acquire appropriate structural and mechanical properties to mimic all types of tissues. Combinations of synthetic and/or natural hydrogels may show an improvement in structural and physical properties with respect to single materials. Moreover, properties can be modulated by preparing hydrogels or blends using single or multiple crosslinking procedures (e.g., physical and ionic ones) [17].

One particular application of hydrogels that could have a significant impact concerns the reconstruction of torn or diseased soft tissues of the osteoarticular system [18,19]. Indeed, the crosslinking of blends of hydrogels can be modulated in order to match the stiffness of the layers that constitute native articular cartilage [20].

In vitro wear evaluation of joint components is mainly performed using the gravimetric method, which is considered the gold standard [21,22]. Currently, advanced imaging techniques are also available, like high-resolution tomography, which allows non-destructive evaluation of the internal structures of the object. Micro-computed tomography (µ-CT) is one of the most versatile non-invasive techniques in the medical field [22,23]. It is able to image internal biological structures without specific sample preparation and could help to overcome the limitation of the gravimetric method. Raman spectroscopy being a non-destructive technique able to provide detailed information on chemical structure and molecular interactions could be a valid alternative to evaluate the composition of hydrogels at molecular level.

Compositions of hydrogels were analyzed with or without embedded graphene oxide (GO, 0.01% *w*/*v*) in order to investigate its influence within the polymeric network. GO exhibits peculiar self-lubricating and anti-wear properties, thanks to its laminar structure which helps to guarantee the lubrication of surfaces [24,25]. The hydrogels were also combined to formulate bilayered structures mimicking the zonal architecture of the articular cartilage (superficial and deep).

Our investigation was aimed at characterizing the wear behavior of hydrogel constructs. The samples were tested using a four-station displacement control knee joint simulator under bovine calf serum. The surface morphology and homogeneity of the worn samples were investigated using µ-CT coupled to roughness measurements.

Moreover, the hydrogels were characterized at a molecular scale with the aim of clarifying their wear behavior in terms of structural composition. For this purpose, Raman spectroscopy was chosen thanks to its suitability as a non-destructive technique to characterize wet samples.

## 2. Materials and Methods

In this study hydrogel constructs were prepared by ionically and physically crosslinking blends made of gellan gum (GG) and poly (ethylene glycol) diacrylate (PEGDA). GG concentration was kept fixed (1.5% *w*/*v*) while PEGDA was used at different concentrations (10 and 15% *w*/*v*), replicating the same composition proposed in [20] and summarized in Table 1.

**Figure 1:**
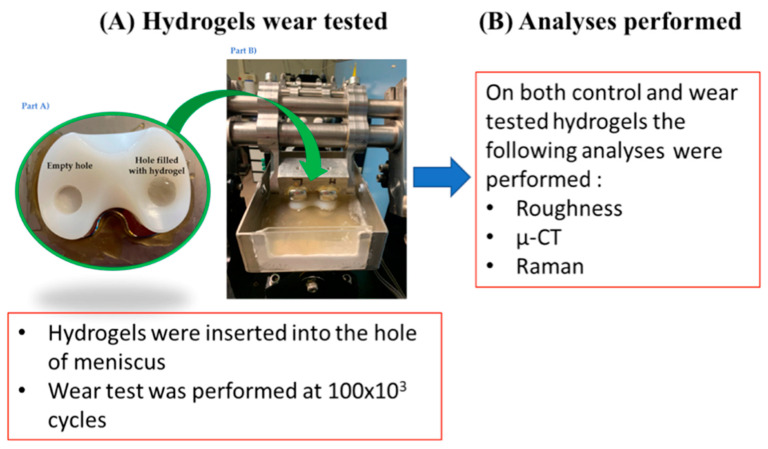
Workflow of the designed protocol: description of hydrogels wear tested. (**A**) Ultra-high-molecular-weight polyethylene (UHMWPE) specimens with the insertion of the hydrogels. A section of the station of the knee simulator with femoral and tibial parts mounted. (**B**) Analyses performed on both control and wear tested hydrogels.

A protocol was properly designed in order to test the hydrogel constructs under a cyclic loading on a knee wear simulator (Figure 1).

### 2.1. Test Specimens

GG (Gelzan™, Merck) was dissolved in deionized water at 65 °C for 1 h (final concentration: 1.5% *w*/*v*) and, after dissolution, PEGDA (Mn: 575, Merck) (final concentrations: 10% *w*/*v* and 15% *w*/*v*) was added under continuous stirring to form the following blends: PEGDA 10% GG 1.5% mimics superior (SUP) layer and PEGDA 15% GG 1.5% for deep (DEEP) layer, respectively [20]. Irgacure2959 (I2959, Merck) initiator was added (final concentration: 0.1% *w*/*v*) in all blends to allow photo-crosslinking. In the case of doped samples, GO (provided by Prof. Nessim, Bar-Ilan University, flake average thickness 4 nm) [26] was added during the last stirring phase at the final concentration of 0.01% *w*/*v*. Summarizing, the samples prepared from blends were DEEP, SUP, DEEP + SUP, DEEP + GO, SUP + GO, and DEEP + SUP + GO.

To perform crosslinking, all blends were poured sequentially into a cylindrical-shape mold, exposed to UV light (λ = 365 nm, 40 mW cm^−2^) for 5 min and incubated with magnesium chloride (MgCl_2_, Merck) for 10 min. The DEEP and SUP formulations (with or without GO) were poured sequentially with a volume ratio of 4:1 and then crosslinked together with the same parameters described above to prepare two types of bilayered structure (DEEP + SUP and DEEP + SUP + GO).

Finally, all cross-linked structures were incubated in DMEM (Dulbecco′s modified Eagle′s medium, Merck, Kenilworth, NJ, USA) for 24 h at 37 °C.

For the mechanical testing, two crosslinked hydrogels for each type were inserted into a hole (diameter 12 mm and height 7 mm) in the UHMWPE meniscal bearings (size 2, Genus mobile bearing, Adler ORTHO, Milan, Italy). Cobalt–chromium TKR femoral components (size 2; Adler ORTHO, Milan, Italy) were fixed onto a metallic holder to fix them onto the knee simulator (Figure 1). A total of 30 specimens were prepared following the same preparation and implantation procedure.

1. Four hydrogels for each batch were used for the mechanical test (test specimens).
2. One hydrogel for each batch was used as control specimen.

### 2.2. Experimental Wear Study Protocol

Mechanical tests were performed using a four-station knee joint simulator (Shore Western, Los Angeles, CA, USA) [27,28,29], meeting the ISO 14,243 requirements, which were originally designed for knee wear testing. The tibial trays and meniscal bearings were mounted in the lower tilting pools. The load was applied vertically to the tibial try oscillating between 168 and 2600 N following a physiological profile. The applied kinematics were in displacement control [30] for the following degrees of freedom:

- flexion/extension angle oscillating between 0 (neutral) and 58° (flexion) synchronously with the load,
- anterior/posterior translation oscillating between 0 (neutral) and 5.2 mm (posterior),
- intra/extra rotation oscillating between 2.1 and 5.7°.

The test duration was set to 100 × 10^3^ cycles at a frequency of 1.0 Hz.

Each station was filled with lubricant (at 37 ± 2 °C) consisting of 25% sterile bovine calf serum (Sigma, St Louis, MI, USA) balanced with deionized water; ethylene-diamine-tetra-acetic acid (20 mmol/dm^3^) was added to minimize precipitation of calcium phosphates.

### 2.3. Roughness Measurements

Measurements of roughness were performed on all wear-tested hydrogels and on all respective controls using an optical profiler (Leica DCM8, Wetzlar, Germany.) in the hydrated state. The average roughness (Ra) was measured on the acquired images converted from three z-stack (scan area 640 × 480 µm^2^) for each sample.

In the present study, only the Ra parameter was measured to characterize the surface roughness. Ra is the average surface roughness obtained as the deviation between the roughness profile and its mean line, or the integral of the absolute value of the roughness profile height over the evaluation length. Scanning operations were performed, acquiring three points at the center of each specimen.

### 2.4. CT Characterization

**Figure 2:**
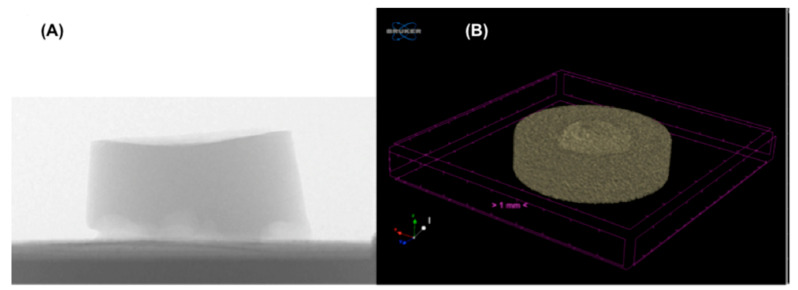
Binarization of reconstructed micro X-ray computed tomography (μ-CT) representative image of SUP hydrogel. (A) A specimen positioned into the μ-CT; (**B**) the reconstructed 3D image of the specimen.

Additional analyses were performed on all specimens tested using micro X-ray computed tomography (μ-CT). In particular, μ-CT characterization was used since it allows global wear volume, local wear distribution, and deformations to be evaluated [31,32]. μ-CT has the potential of obtaining quantitative three-dimensional information of the entire object geometry in a non-destructive and non-contact way [22]. Microscopic examination of all hydrogel constructs was performed using the non-invasive X-ray detection μ-CT (Skyscan 1072, Bruker Corp., MicroCT unit, Kontich, Belgium) in order to assess the uniformity of the molecular structure. The exposure time was set at 5936 ms using a beam of 50 kV −197 µA. In Figure 2, the set-up of the specimens’ acquisition using μ-CT is shown.

### 2.5. Raman Characterization

Raman spectra were recorded on a Bruker MultiRam FT-Raman spectrometer equipped with a cooled Ge-diode detector. The excitation source was a Nd^3+^-YAG laser (1064 nm) in the backscattering (180°) configuration. The focused laser beam diameter was about 100 μm and the spectral resolution 4 cm^−1^. Laser power at the samples was about 180 mW without decomposition. The spectra were recorded non-invasively on wet samples as well as after drying, to inspect more confidently the spectral range near 1600–1650 cm^−1^ (where water has a broad Raman band). Only specimens free from GO were analyzed due to the strong spectral background observed in GO-added samples (Figure S1, Supplementary Material), which did not allow any reliable characterization of the polymeric component under the used experimental conditions.

Three spectra at least were recorded in three different positions of each sample. GG and uncrosslinked PEGDA as well as photo-crosslinked 10% *w*/*v* and 15% *w*/*v* PEGDA were analyzed.

Average spectra normalized to the intensity of the CH_2_ bending band at about 1470 cm^−1^ were reported. The relative percentage of C=C bonds in the hydrogels was obtained by comparing the peak height at 1640 cm^–1^ (attributed to C=C stretching), which was used as a measure of the degree of crosslinking, according to the following equation:

(1) $$\mathrm{Relative}\%\;\mathrm{of}\;C=C\;\mathrm{bonds}\;=\;\frac{A_{1640\;\mathrm{hydrogel}}}{A_{1640\;\mathrm{starting}\;\mathrm{PEGDA}\;}}\times 100$$

where A_1640 hydrogel_ and A_1640 starting PEGDA_ were the areas of the band at about 1640 cm^−1^ in the spectra of the hydrogel and starting PEGDA, respectively, as calculated from the normalized spectra.

## 3. Results

### 3.1. Roughness Results

**Figure 3:**
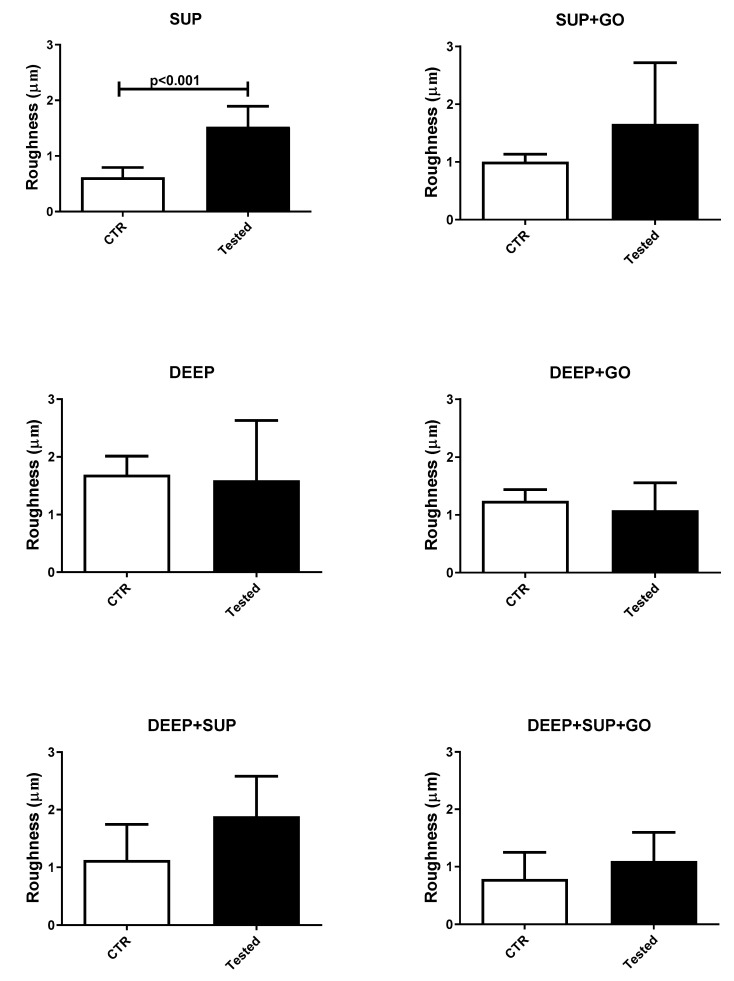
Average roughness values of the hydrogels under study: control (CTR) and tested samples (SUP, DEEP, DEEP + SUP without or with (+) GO).

**Figure 4:**
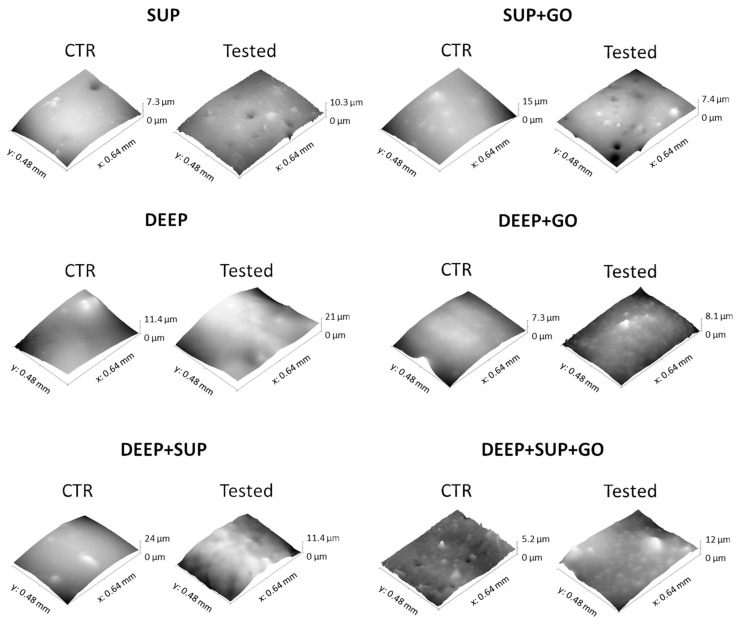
Tridimensional reconstruction of the hydrogel surface under study: control (CTR) and tested samples (SUP, DEEP, DEEP+SUP without or with (+) GO).

The average roughness values measured for the hydrogels involved in the study, compared with their respective controls (not undergoing the wear test), are shown in Figure 3 and Figure 4.

Only the SUP hydrogel formulation (i.e., the one containing the lowest PEGDA percentage, 10%) showed a statistically significant increase in roughness upon wear test. The hydrogels made of 15% *w*/*v* of PEGDA showed an optimal resistance to wear, and the presence of GO contributed to qualitatively reducing roughness modifications.

### 3.2. CT Results

Results of µ-CT analysis showed a different behavior between all the specimens.

**Figure 5:**
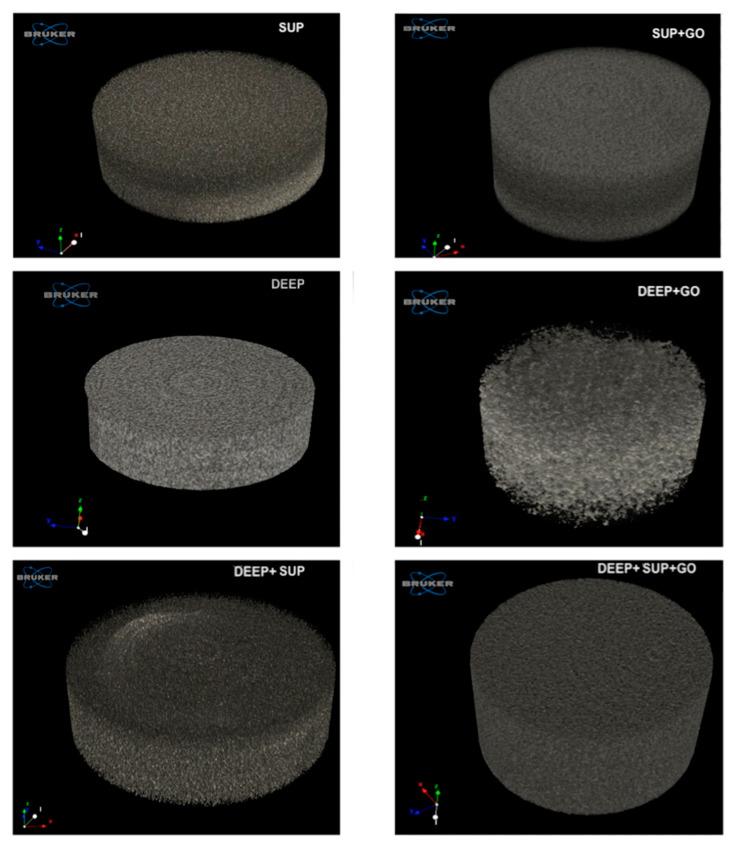
Representative images of binarized and reconstructed μ-CT images for each specimen (SUP; SUP + GO; DEEP; DEEP + GO; DEEP + SUP; DEEP + SUP + GO).

In Figure 5 all the tested hydrogels are shown, with their respective 3D reconstruction.

In the hydrogels DEEP, DEEP + SUP, and DEEP + SUP + GO, a uniformly distributed density was observed, indicating a more homogeneous crosslinking degree. All these hydrogels contain 15% PEGDA, which independently from the presence (DEEP + SUP + GO) or absence (DEEP, DEEP + SUP) of GO contributes to form a structure with uniform density.

### 3.3. Raman Results

As an example, Figure S2, Supplementary Material, shows the average Raman spectra of uncrosslinked PEGDA as well as photo-crosslinked 10% and 15% *w*/*v* PEGDA (wet samples). Not unexpectedly, as observable from Figure S3, Supplementary Material, wet and dried samples were different in the wavenumber position, relative intensity, and resolution of several bands, as detailed in Table S1, Supplementary Material, where the main assignments of Raman bands of PEGDA are reported according to the literature [33,34,35].

Comparing the PEGDA solution to the photo-crosslinked 10% and 15% PEGDA hydrogels (DEEP + SUP, SUP, and DEEP), the bands assignable to diacrylate groups (Table S1, Supplementary Material) progressively decreased in intensity without disappearing.

**Figure 6:**
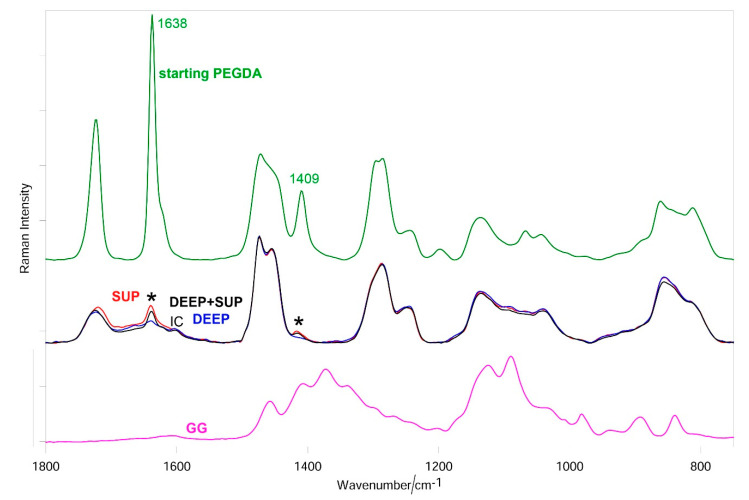
Average Raman spectra (normalized to the intensity of the CH_2_ bending band at about 1470 cm^−1^) of wet SUP, DEEP, and DEEP + SUP hydrogels; the spectra of starting PEGDA and GG are reported for comparison. The main bands that decrease in intensity upon photo-crosslinking (assignable to diacrylate groups) are indicated with an asterisk. The band at 1601 cm^−1^ is assignable to Irgacure2959 (IC).

Figure 6 shows the average Raman spectra of SUP, DEEP, and DEEP + SUP hydrogels; the spectra of uncrosslinked PEGDA and GG are reported for comparison. As can be easily seen, the spectra of the hydrogels are dominated by the bands of PEGDA and no spectral features of GG were detected according to its low content in the blend. Going from starting PEGDA to SUP and DEEP hydrogels, the bands due to the diacrylate groups progressively weakened but did not disappear. In the DEEP + SUP sample the diacrylate bands had intermediate intensities between SUP and DEEP hydrogels, although more similar to the SUP specimen. Analogous trends were observed in the spectra of the samples after drying (Figure S4, Supplementary Material).

**Figure 7:**
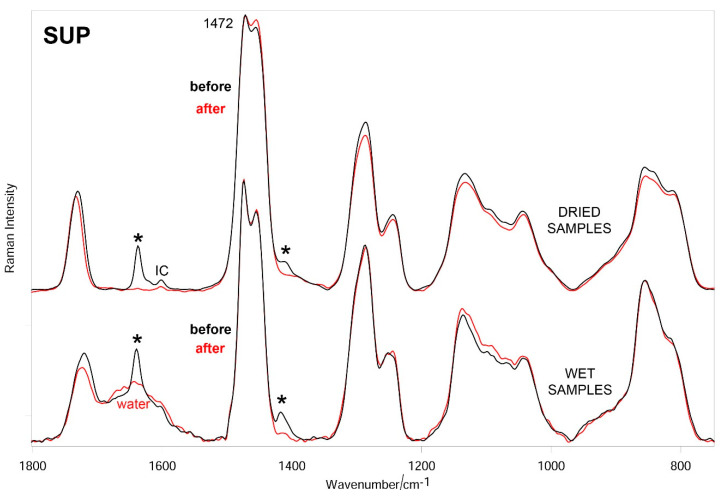
Average Raman spectra (normalized to the intensity of the CH_2_ bending band at about 1470 cm^−1^) of SUP-GO hydrogels before and after testing (wet and dried samples). The main bands that decrease in intensity upon testing are indicated with an asterisk. The band at 1601 cm^−1^ is assignable to Irgacure2959 (IC).

**Figure 8:**
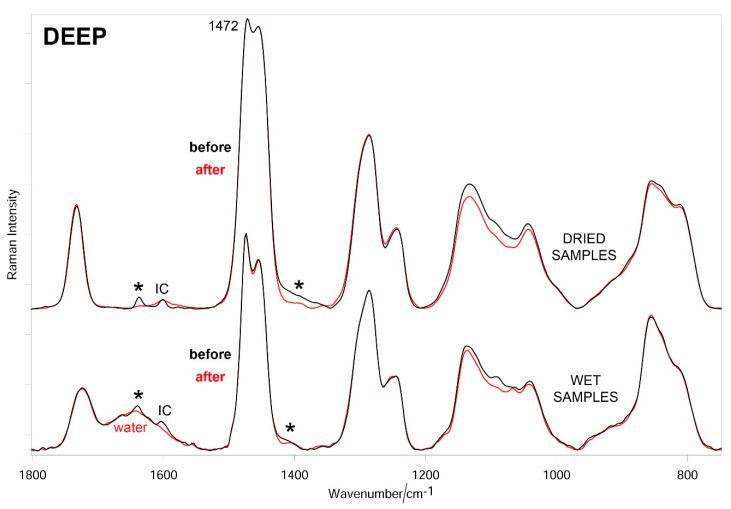
Average Raman spectra (normalized to the intensity of the CH_2_ bending band at about 1470 cm^−1^) of DEEP hydrogels before and after testing (wet and dried samples). The main bands that decrease in intensity upon testing are indicated with an asterisk. The band at 1601 cm^−1^ is assignable to Irgacure2959 (IC).

**Figure 9:**
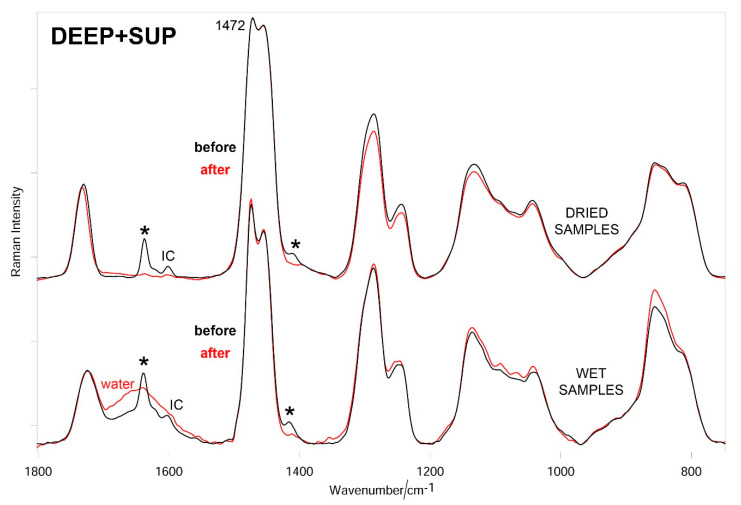
Average Raman spectra (normalized to the intensity of the CH_2_ bending band at about 1470 cm^−1^) of DEEP + SUP hydrogels before and after testing (wet and dried samples). The main bands that decrease in intensity upon testing are indicated with an asterisk. The band at 1601 cm^−1^ is assignable to Irgacure2959 (IC).

Figure 7, Figure 8 and Figure 9 show the average Raman spectra of SUP, DEEP, and DEEP + SUP hydrogels before and after testing (wet and dried samples), respectively.

**Figure 10:**
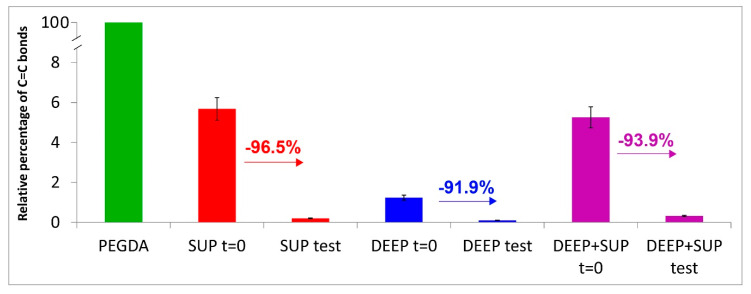
Values (average ± standard deviation) of the relative percentage of C=C bonds in the hydrogels under study, calculated according to Equation (1).

In all the cases, a significant decrease in intensity of the bands due to diacrylate groups was observed upon testing both in wet and dried samples. From a more quantitative point of view, the trend of the relative percentage of C=C bonds in the hydrogels under study was calculated according to Equation (1). Figure 10 confirms these qualitative results.

## 4. Discussion

Osteoarthritis is a term commonly used to describe the clinical and pathological outcomes of an active process that involves cartilage destruction, subchondral bone thickening, and results in the structural and functional failure of a joint [36,37]. Often, people suffering from knee pain are affected by OA and the way to “solve” this problem is the implantation of a knee prosthesis. The number of revisions of total knee arthroplasties is rising exponentially with a projected increase of 601% in 2030 [38,39]. For this reason, it is very important to have a systematic approach to dealing with patients presenting painful TKA. In most cases, however, other treatment options are exhausted before an artificial knee is implanted. Articular cartilage is a particular tissue that lacks a blood supply to support repair and remodeling [40,41]. Cartilage tissue engineering offers an alternative method for treating arthritis [41].

This study aimed to characterize the wear behavior of hydrogel constructs that were developed to mimic both the properties of the superficial and deep layers of human articular cartilage, as reported in [20]. PEGDA and GG were used at different concentrations and double-crosslinked with UV and ions. The developed constructs were tested using a four-station displacement control knee joint simulator under bovine calf serum.

Roughness and µ-CT measurements showed different wear behaviors for the samples under study. In particular, the samples containing photo-crosslinked 10% PEGDA showed a worse behavior than those made with 15% of PEGDA both in terms of roughness increase and loss of uniformly distributed density. Raman measurements allowed more insights to be gained into these trends. Unreacted C=C bonds were detected in all the hydrogels after crosslinking before the wear test. In the DEEP-GO samples the bands assignable to diacrylate groups were observed with the lowest relative intensities (Figure 6); the relative percentage of C=C bonds calculated according to Equation (1) confirmed these qualitative trends (Figure 10). In the control hydrogels, this parameter was found to decrease along the series SUP > DEEP+SUP > DEEP in the absence of GO that showed high spectral background. In other words, the DEEP samples that upon photo-polymerization attained the highest degree of crosslinking were more resistant. Therefore, it is not surprising that they showed a better wear behavior than the other samples at roughness and µ-CT analyses, since it is well known that the degree of crosslinking influences polymer degradation [42,43]

To the author’s knowledge, this is the first study in which hydrogels for cartilage applications were characterized using a µ-CT technique. A study by Douglas et al. [44] analyzed, using µ-CT, a generation of injectable hydrogel-ceramic composites of GG and different bioglasses. Serafim and co-workers [45] report about application of hydrogel coatings for abdominal wall repair. These authors considered the potential of hydrogels to mimic the cellular microenvironment and they investigated the surface morphology and homogeneity of their constructs using µ-CT. Datta [46] and colleagues analyzed the degradation behavior of some hydrogel constructs using the µ-CT technique.

The Raman spectra of the tested hydrogels (Figure 7, Figure 8 and Figure 9) showed the loss of diacrylate groups in all the samples. This behavior could be explained by considering that unreacted PEGDA underwent washout in a bovine calf serum aqueous environment. As can be seen from Figure 10, the relative percentage of C=C bonds significantly decreased in all the hydrogels. As previously observed, it is not surprising that the percentage decrease in this parameter varies along the same series as reported above, i.e., SUP > DEEP+SUP > DEEP, further confirming that the degree of photo-crosslinking of the starting materials plays a key role in determining their wear behavior. However, the strong spectral background observed in specimens with GO limited the analysis since these samples could not be evaluated using Raman.

Noted limitations were typical for wear simulation. The number of simulated hydrogels available for analysis was restricted by the number of stations incorporated in the knee joint wear simulator. Moreover, hydrogels were simulated only in vitro and no comparison with their in vivo behavior was done. Finally, the gravimetric method which is considered the gold standard for wear measurements could not be used with hydrogels since they are prone to dehydrate and lose their original weight.

## 5. Conclusions

In conclusion, all the techniques used to evaluate the hydrogels evidenced that the different degree of crosslinking after photo-polymerization influenced their resistance after a wear test that mimicked a patient that had walked for approximately three months. The higher concentration of PEGDA (15%) contributed to reducing the roughness and the 3D structure changes of the hydrogels after the wear test. Raman spectroscopy proved a suitable technique to characterize the hydrogel constructs under wet conditions, i.e., with no sample manipulation needed. Spectroscopic measurements showed that the degree of photo-crosslinking of the starting material strongly influences its wear behavior. These data suggest that the combination of these different techniques have contributed to a deep characterization of the hydrogels after a wear test by considering at the same time not only the superficial roughness but also the whole 3D structure and their composition. This approach represents a new interesting protocol to have a complete and deep evaluation of 3D hydrogels.

## Figures and Tables

**Table 1:** Description of materials content and study groups.

Materials Content	Study Groups							
	SUP	SUP + GO	DEEP	DEEP + GO	DEEP + SUP		DEEP + SUP + GO	
**GG**	1.5%	1.5%	1.5%	1.5%	1.5%	1.5%	1.5%	1.5%
**PEGDA**	10%	10%	15%	15%	15%	10%	15%	10%
**GO**	-	0.01%	-	0.01%	-	-	0.01%	0.01%

## Data Availability

Not applicable.

## Supplementary Materials

The following are available online at https://www.mdpi.com/1996-1944/14/2/428/s1, Figure S1: Raman spectrum of DEEP + GO/SUP + GO; the strong spectral background does not allow any reliable characterization of the polymeric phase, Figure S2: Average Raman spectra of starting PEGDA as well as photo-crosslinked 10% and 15% PEGDA hydrogels (wet samples). The spectra are normalized to the intensity of the CH_2_ bending band at about 1470 cm^−1^. The main bands that decrease in intensity upon photo-crosslinking (assignable to diacrylate groups) are indicated with an asterisk, Figure S3: Average Raman spectra of photo-crosslinked 10% PEGDA hydrogel (dried and wet samples). The spectra are normalized to the intensity of the CH_2_ bending band at about 1470 cm^−1^, Figure S4: Average Raman spectra (normalized to the intensity of the CH_2_ bending band at about 1470 cm^−1^) of SUP, DEEP and DEEP + SUP hydrogels after drying. The main bands that decrease in intensity upon photo-crosslinking (assignable to diacrylate groups) are indicated with an asterisk. The band at 1601 cm^−1^ is assignable to Irgacure2959 (IC), Table S1: Wavenumbers (cm^−1^) and assignments [34,35,36] of the main Raman bands of PEGDA and photo-crosslinked 10% PEGDA hydrogel (dried and wet samples). The main bands that decrease in intensity upon photo-crosslinking are indicated in bold characters; they are prevalently assignable to the diacrylate groups that are involved in crosslinking.

## Abbreviations

GG	gellan gum
PEGDA	poly (ethylene glycol) diacrylate
GO	graphene oxide
DMEM	Dulbecco′s modified Eagle′s medium
MgCl_2_	magnesium chloride
SUP	superior layer
DEEP	deep layer
DEEP	deep layer without graphene oxide
SUP	superior layer without graphene oxide
DEEP + SUP	deep and superior layers both without graphene oxide
DEEP + GO	graphene oxide-embedded deep layer
SUP + GO	graphene oxide-embedded superior layer
DEEP + SUP + GO	deep and superior layers both with embedded graphene oxide
