# Supplementary material for: Wear Behavior Characterization of Hydrogels Constructs for Cartilage Tissue Replacement
Source: Materials (Basel). 2021 Jan 16;14(2):428. doi: 10.3390/ma14020428 (PMC7830039; doi:10.3390/ma14020428)
Supplement: Supplementary file 1 [file materials-14-00428-s001.pdf]

materials

MDPI

Supplementary Material

Wear Behavior Characterization of Hydrogels Constructs for Cartilage Tissue Replacement

Saverio Affatato [1,*], Diego Trucco [2,3,4], Paola Taddei [5], Lorenzo Vannozzi [3,4], Leonardo Ricotti [3,4], Gilbert Daniel Nessim [6] and Gina Lisignoli [2]

1. IRCSS Istituto Ortopedico Rizzoli, Laboratorio di Tecnologia Medica, 40136 Bologna, Italy
2. IRCSS Istituto Ortopedico Rizzoli, SC Laboratorio di Immunoreumatologia e Rigenerazione Tissutale, 40136 Bologna, Italy; diego.trucco@santannapisa.it (D.T.); gina.lisignoli@ior.it (G.L.)
3. The BioRobotics Institute, Scuola Superiore Sant'Anna, Piazza Martiri della Libertà 33, 56127 Pisa, Italy; lorenzo.vannozzi@santannapisa.it (L.V.); leonardo.ricotti@santannapisa.it (L.R.)
4. Department of Excellence in Robotics & AI, Scuola Superiore Sant'Anna, Piazza Martiri della Libertà 33, 56127 Pisa, Italy
5. Dipartimento di Scienze Biomediche e Neuromotorie, Università di Bologna, Via Belmeloro 8/2, 40126 Bologna, Italy; paola.taddei@unibo.it
6. Department of Chemistry, Bar-Ilan Institute for Nanotechnology and Advanced Materials, Bar-Ilan University, Ramat Gan 52900, Israel; Gilbert.Nessim@biu.ac.il

* Correspondence: affatato@tecno.ior.it; Tel.: +39-051-6366864

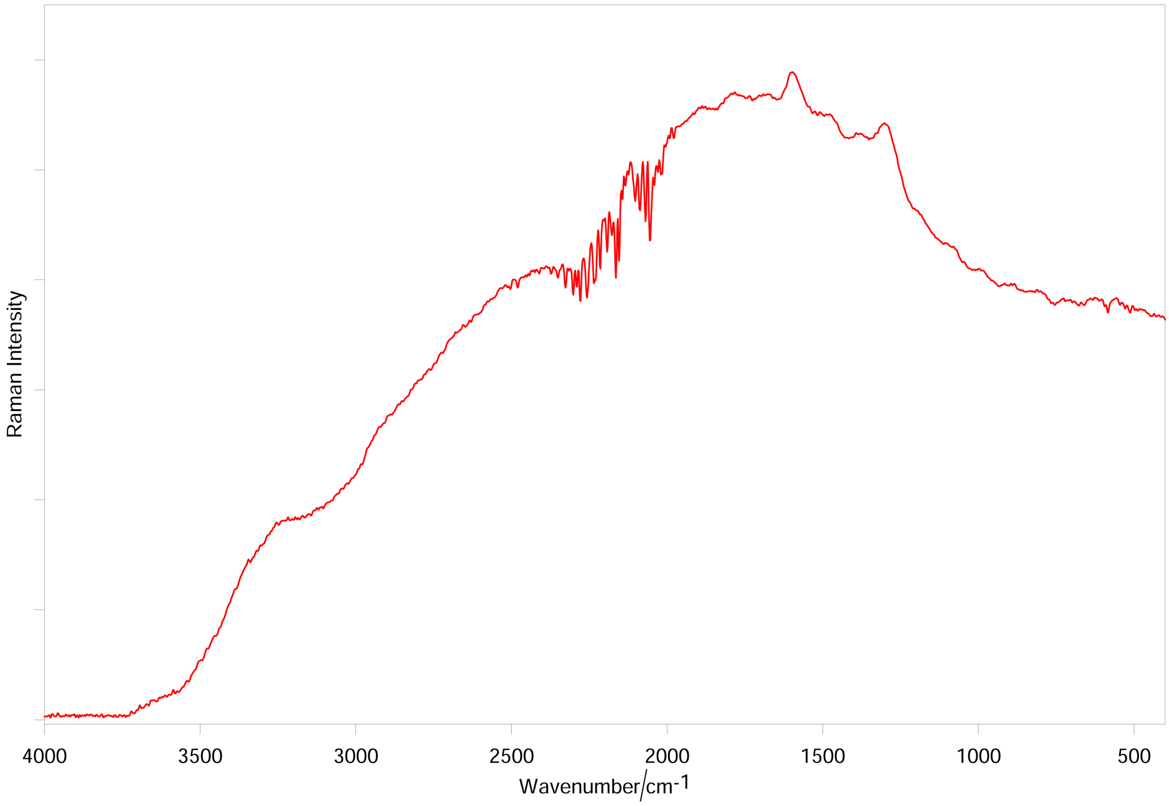

Figure S1. Raman spectrum of DEEP+GO/SUP+GO; the strong spectral background does not allow any reliable characterization of the polymeric phase.

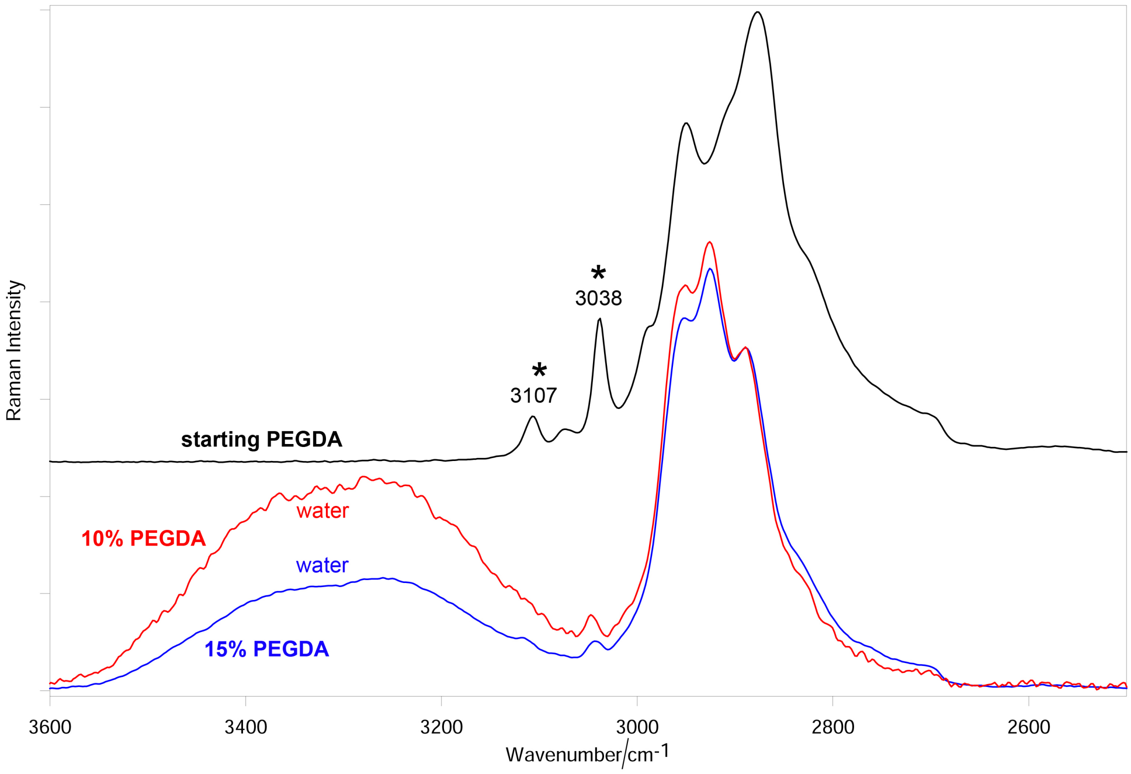

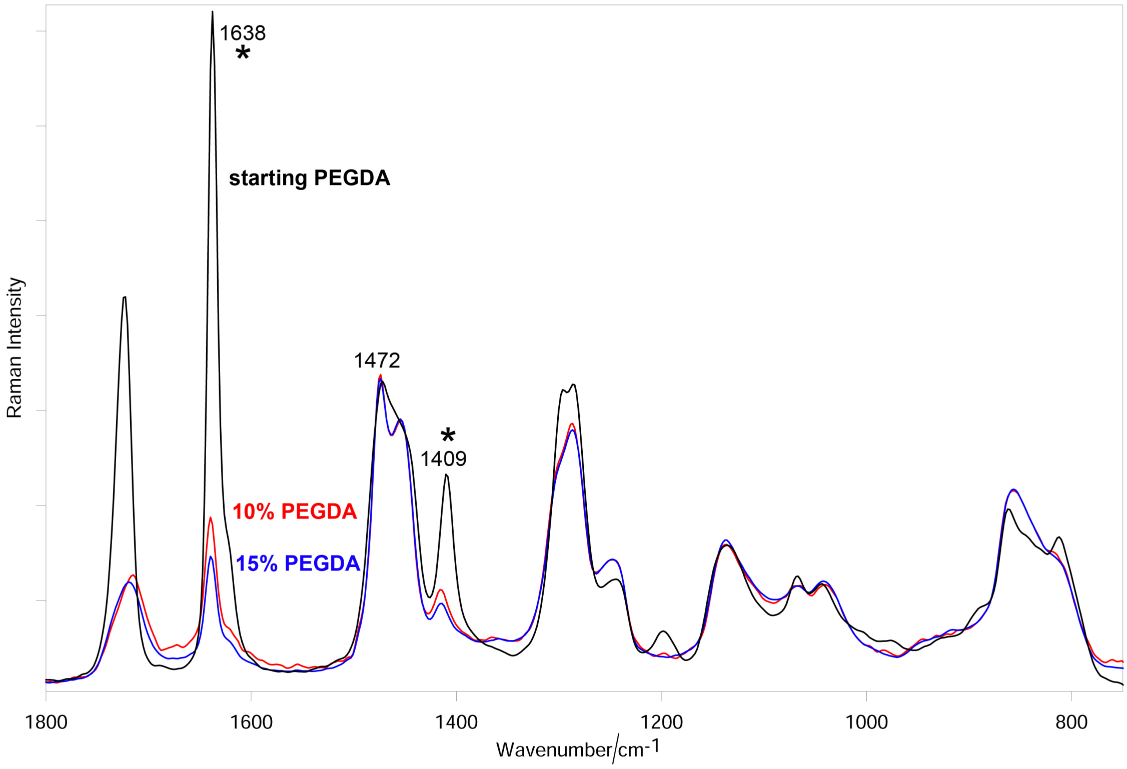

Figure S2. Average Raman spectra of starting PEGDA as well as photo-crosslinked 10% and 15% PEGDA hydrogels (wet samples). The spectra are normalized to the intensity of the CH_2 bending band at about 1470 cm^{-1}. The main bands that decrease in intensity upon photo-crosslinking (assignable to diacrylate groups) are indicated with an asterisk.

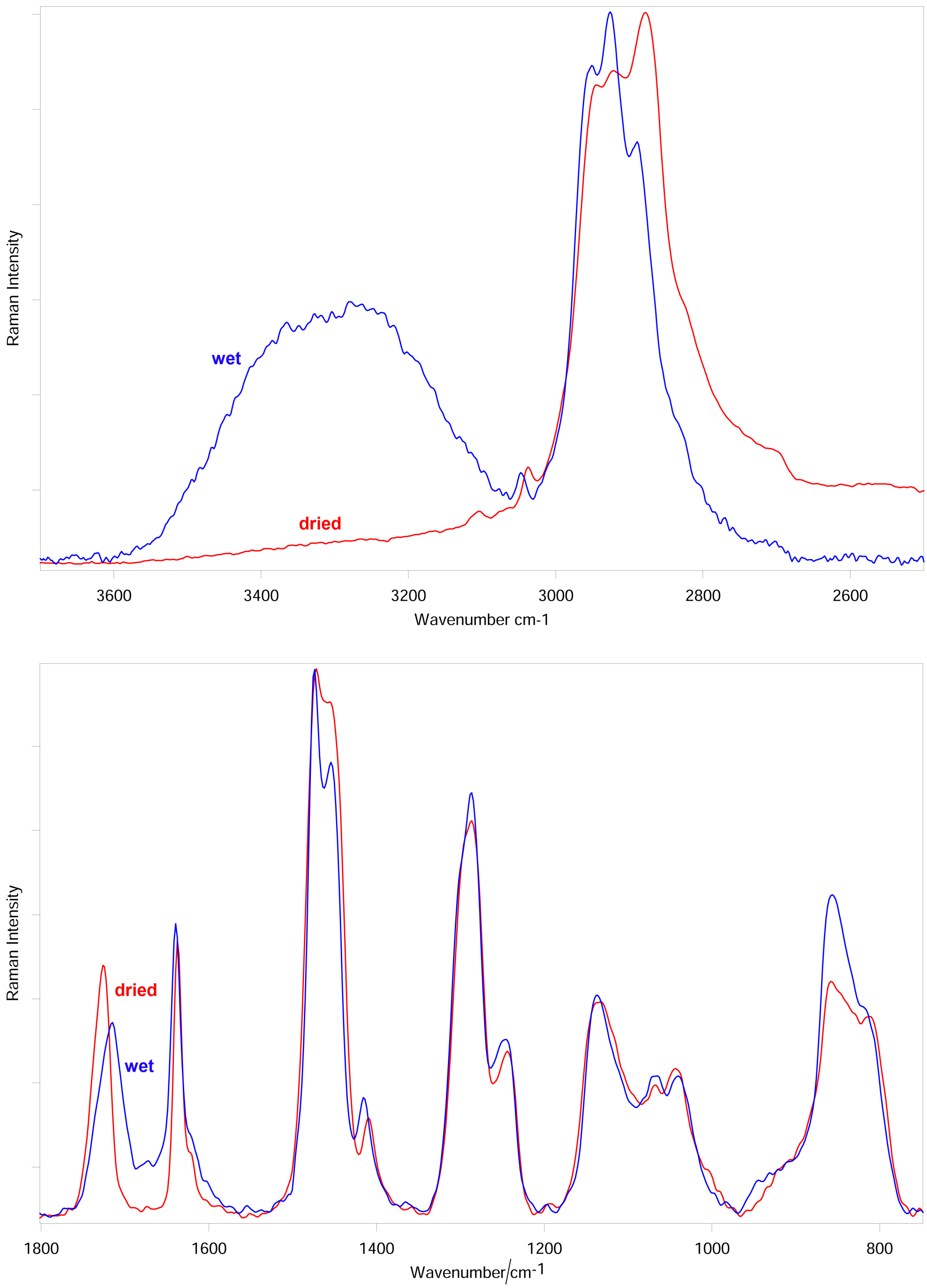

Figure S3. Average Raman spectra of photo-crosslinked 10% PEGDA hydrogel (dried and wet samples). The spectra are normalized to the intensity of the CH_2 bending band at about 1470 cm^{-1}.

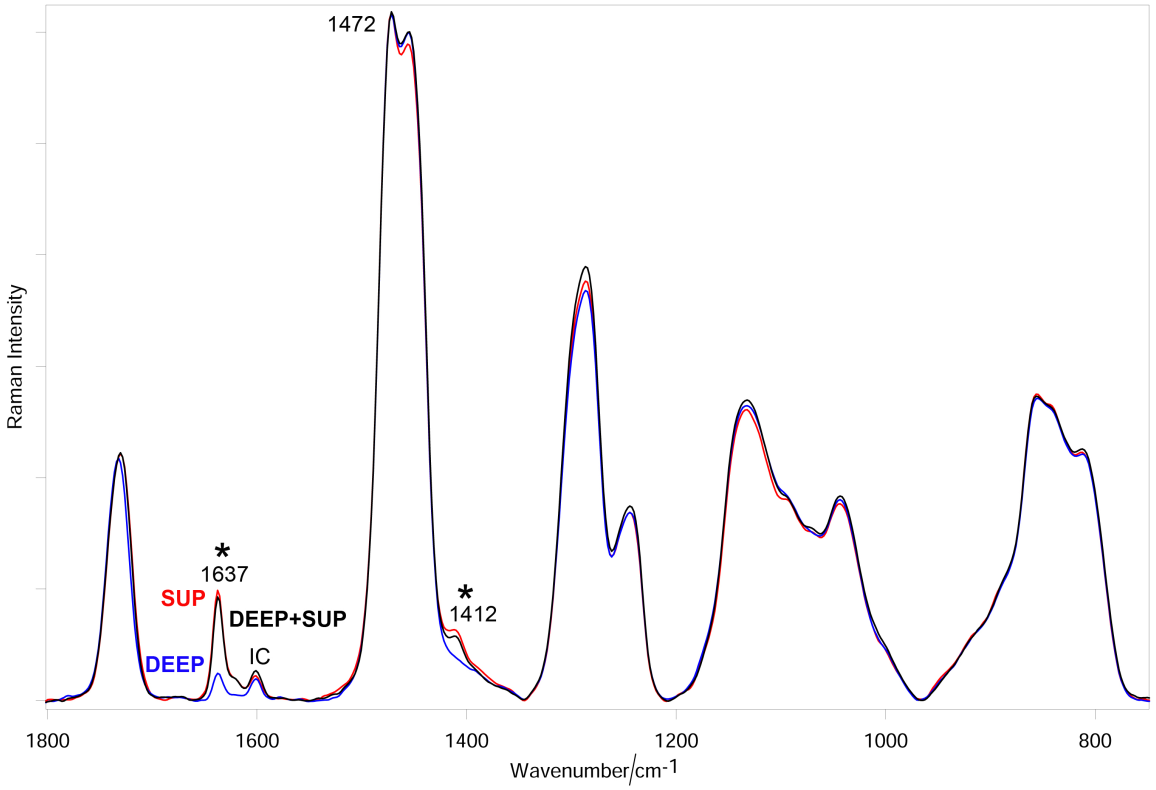

Figure S4. Average Raman spectra (normalized to the intensity of the CH_2 bending band at about 1470 cm^{-1}) of SUP, DEEP and DEEP+SUP hydrogels after drying. The main bands that decrease in intensity upon photo-crosslinking (assignable to diacrylate groups) are indicated with an asterisk. The band at 1601 cm^{-1} is assignable to Irgacure2959 (IC).

Table S1. Wavenumbers (cm^{-1}) and assignments [34-36] of the main Raman bands of PEGDA and photo-crosslinked 10% PEGDA hydrogel (dried and wet samples). The main bands that decrease in intensity upon photo-crosslinking are indicated in bold characters; they are prevalently assignable to the diacrylate groups that are involved in crosslinking.

Wavenumber (cm^{-1})			Assignments
PEGDA	crosslinked 10% PEGDA, dried sample	crosslinked 10% PEGDA, wet sample	
		3360-3250 (broad)	water OH stretching
**3107**	**3106**		**=CH stretching**
**3038**	**3037**	**3046**	**=CH stretching**
2950	2943	2953	alkyl CH stretching
	2923	2926	alkyl CH stretching
2877	2877	2890	alkyl CH stretching
1723	1728	1716	C=O stretching
**1638**	**1637**	**1639**	**C=C stretching**

1472	1472	1474	CH_2 bending
1458	1455	1454	CH_3 antisymmetric bending and CH_2 scissoring
**1409**	**1410**	**1415**	**=CH_2 twisting or wagging**
1286	1287	1287	ester group
1246	1246	1250	ester group
1198			
1136	1133	1137	skeletal stretching
1068	1069	1071	skeletal stretching
1043	1043	1042	skeletal stretching
862	857	857	
813	813	816	
